# Silicon moderated the K deficiency by improving the plant-water status in sorghum

**DOI:** 10.1038/srep22882

**Published:** 2016-03-10

**Authors:** Daoqian Chen, Beibei Cao, Shiwen Wang, Peng Liu, Xiping Deng, Lina Yin, Suiqi Zhang

**Affiliations:** 1 State Key Laboratory of Soil Erosion and Dryland Farming on the Loess Plateau, Institute of Soil and Water Conservation, Northwest A&F University, Yangling, Shaanxi, 712100, China; 2 College of Natural Resources and Environment, Northwest A&F University, Yangling, Shaanxi, 712100, China; 3 College of Life Sciences, Northwest A&F University, Yangling, Shaanxi, 712100, China; 4 Department of Plant Protection, Shandong Agricultural University, Tai’an, Shandong, 271018, China

## Abstract

Although silicon (Si) has been widely reported to alleviate plant nutrient deficiency, the underlying mechanism in potassium (K) deficiency is poorly understood. In this study, sorghum seedlings were treated with Si under a K deficiency condition for 15 days. Under control conditions, plant growth was not affected by Si application. The growth and water status were reduced by K-deficient stress, but Si application significantly alleviated these decreases. The leaf gas exchanges, whole-plant hydraulic conductance (K_plant_), and root hydraulic conductance (*Lp*_r_) were reduced by K deficiency, but Si application moderated the K-deficiency-induced reductions, suggesting that Si alleviated the plant hydraulic conductance. In addition, 29% of Si-alleviated transpiration was eliminated by HgCl_2_ treatment, suggesting that aquaporin was not the primary cause for the reversal of plant hydraulic conductance. Moreover, the K^+^ concentration in xylem sap was significantly increased and the xylem sap osmotic potential was decreased by Si application, suggesting that the major cause of Si-induced improvement in hydraulic conductance could be ascribed to the enhanced xylem sap K^+^ concentration, which increases the osmotic gradient and xylem hydraulic conductance. The results of this study show that Si mediates K^+^ accumulation in xylem, which ultimately alleviates the plant-water status under the K-deficient condition.

Potassium (K) is the most abundant cation in plants. It is involved in various important cellular processes, such as the stabilization of protein synthesis, activation of enzymes, and neutralization of negative charges on proteins^1^,^2^. In addition, it also plays a key role in osmotic processes that contribute to cellular turgor, photosynthesis, and transpiration^3^. K deficiency can disturb these activities directly, and inhibit plant growth and development^2^,^4^,^5^. K is also essential for high-yield crop production, and it can be a limiting factor for such crops under certain environmental conditions^6^,^7^. The uptake of silicon (Si), which is one of the most abundant elements in the Earth’s crust, is beneficial for increasing plant resistance to various stresses^8^,^9^,^10^. Si application has also been widely reported to alleviate plant nutrient deficiency, including alleviating K deficiency^4^,^11^,^12^,^13^.

Compared with the benefits of Si under other stresses, the performance and underlying mechanism of Si in alleviating K deficiency has rarely been investigated^4^. To date, only one study has reported that Si application alleviated K-deficiency-induced growth inhibition in soybean. Several studies also reported that Si can affect tissue K concentration under salt stress conditions^14^,^15^. These previous studies suggested that Si can influence the K absorption and/or distribution under certain conditons. K deficiency disturbs plant growth and development in various ways. It is not clear if there are other ways in which Si participates in regulating K deficiency. A better understanding of the interaction between Si application and K deficiency will provide important information about Si and K fertilizer use in crop production.

The Plant-water status involves the balance of the difference in water uptake through the roots and transpiration in leaves; maintaining water status is the basis of metabolic activities in tissues^15^,^16^. K^+^ is involved in regulating the plant-water status in various ways. First, K^+^ plays an important role in stomatal opening and closing, which controls leaf transpiration and plant-water status^17^,^18^. Second, at the whole-plant level, K^+^ accumulation in the root xylem vessels is also involved in osmotic water absorption^19^. Third, several recent reports have suggested that xylem K^+^ plays a critical role in modifying xylem hydraulic conductance and water relations in plants^5^,^20^. In contrast, it has been widely reported that K deficiency affects the plant-water status in different ways. Severe K deficiency generally induces stomatal closure and lowers transpiration rates^21^. As the main osmotic solute, K deficiency also affects the osmotic potential gradient and decreases water uptake^20^,^22^. K deficiency was found to reduce aquaporin activity, thereby suppressing root hydraulic conductance and water supply to the stem and leaf transpiration^21^. K fertilizer, however, was found to increase plant hydraulic conductance and transpiration^20^. In sum, K is involved in regulating the plant water status, and severe K deficiency causes tissue dehydration^21^.

There are no case studies showing that Si application affects the plant-water status under K deficiency stress, but Si was found to be involved in regulating the plant-water status in response to other stresses. Romero-Aranda *et al.*^23^ demonstrated that Si application enhanced the tomato leaf water content under salt stress. Recently, Liu *et al.*^24^,^25^ reported that enhanced root hydraulic conductance by aquaporin regulation is responsible for Si-alleviated plant-water status under drought and salt stresses. Under normal growth conditions, Si application was also found to affect the plant-water status^15^ and up-regulate aquaporin gene *(PIPs)* expression^25^. In addition, the potential effect of Si application on K^+^ absorption and distribution may be involved in regulating the plant water status. Therefore, the close coupling between K deficiency and Si application on the plant-water status suggests that Si may be involved in regulating the plant water-status under K-deficiency conditions.

The aim of this study was to determine whether or not Si improves the plant-water status under K-deficiency conditions. We hypothesized that K deficiency induces a decrease in plant hydraulic conductance, which decreases the water supply for leaf transpiration and plant-water status. Si application increases plant hydraulic conductance, possibly favoring higher gas exchange rates and photosynthesis, thus alleviating the K deficiency. To accomplish the objective of this study, the effect of Si application on leaf relative water content (LRWC), plant transpiration, whole-plant hydraulic conductance (K_plant_) and root hydraulic conductance (*Lp*_r_), and the effect of aquaporin on transpiration and xylem sap K^+^ concentration were measured under K-deficiency and Si application conditions. Moreover, the expression of aquaporin and K^+^-related genes were also investigated.

## Results

### Si application alleviated the K deficiency in sorghum and improved the plant-water status

**Figure 1:**
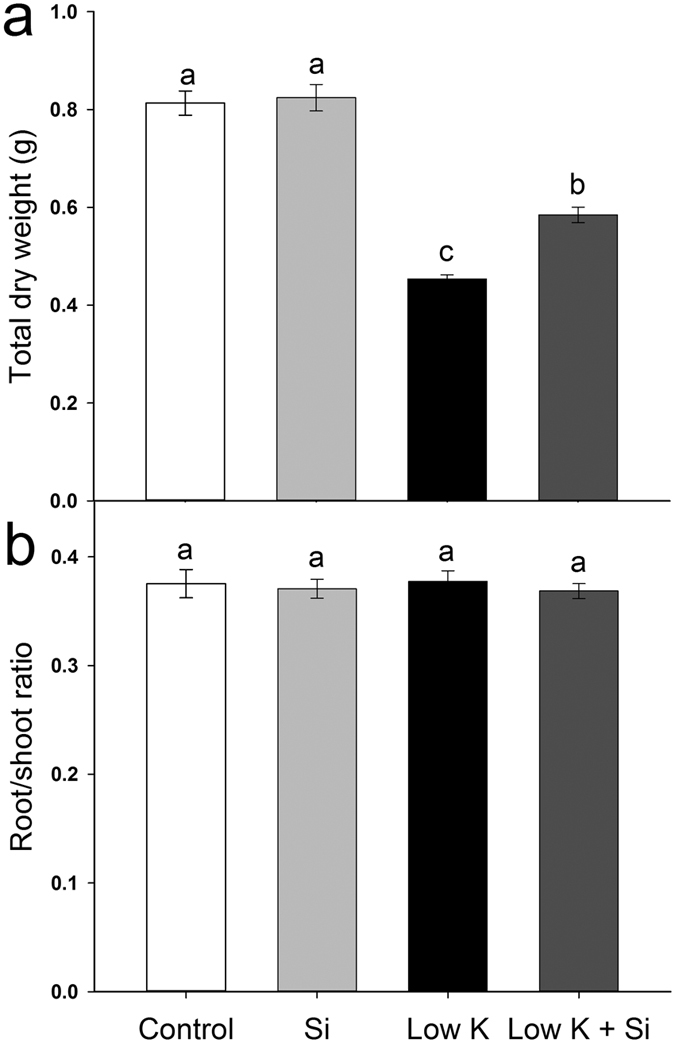
Effects of silicon (Si, 1 mM) on the plant growth of sorghum plants grown under control (3 mM KCl) and low K (0.05 mM KCl) conditions. (**a**) Total dry weight, (**b**) root/shoot ratio. All parameters were measured after 15 days of treatment. Values are presented as the mean ± SE (n = 24). Different letters in one measure indicate statistically significant differences at *P* < 0.05.

**Figure 2:**
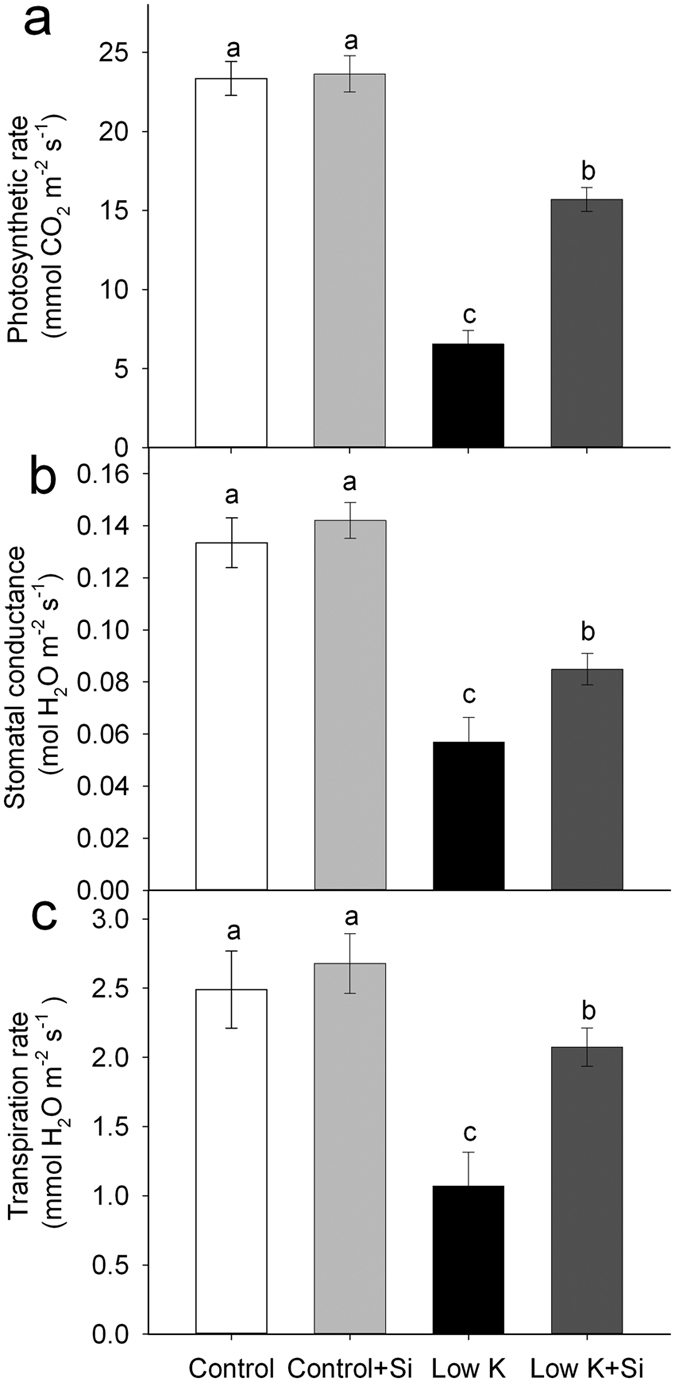
Effects of silicon (Si, 1 mM) on photosynthetic rate (**a**), stomatal conductance (**b**) and transpiration rate (**c**) of sorghum plants grown under control (3 mM KCl) and low K (0.05 mM KCl) conditions. All parameters were measured after 15 days of treatment. Values are presented as the mean ± SE (n = 6). Different letters in one measure indicate statistically significant differences at *P* < 0.05.

Extreme K deficiency inhibits plant growth^26^. In the present study, Si application did not affect the sorghum seedling growth under control conditions. However, K deficiency significantly decreased plant growth after 15 days incubation and Si application alleviated the K-deficiency-induced growth inhibition (Fig. 1a, Supplementary Fig. S1). The dry weight of Si-treated plants was 29% (*p* < 0.05) higher than that of untreated plants under K deficiency conditions. At the same time, the root/shoot ratio was not affected by either K deficiency or Si application (Fig. 1b). These results showed that Si application enhanced plants’ K deficiency tolerance. Similar to the change in plant dry weight, the stomatal conductance, transpiration rate, and photosynthetic rate were also found to be decreased under K deficiency in various plant species^21^,^27^,^28^. In this study, the leaf gas exchange was not influenced by Si under control conditions, but it was significantly inhibited by K deficiency. Si application alleviated the K-deficiency-induced reduction in the photosynthetic rate, stomatal conductance and transpiration rate (Fig. 2). In Si-treated plants, the leaf photosynthetic rate, stomatal conductance and transpiration rate were 139% (*p* < 0.05), 49% (*p* < 0.05) and 94% (*p* < 0.05) higher than that of plants without Si application under K-deficiency conditions (Fig. 2).

**Figure 3:**
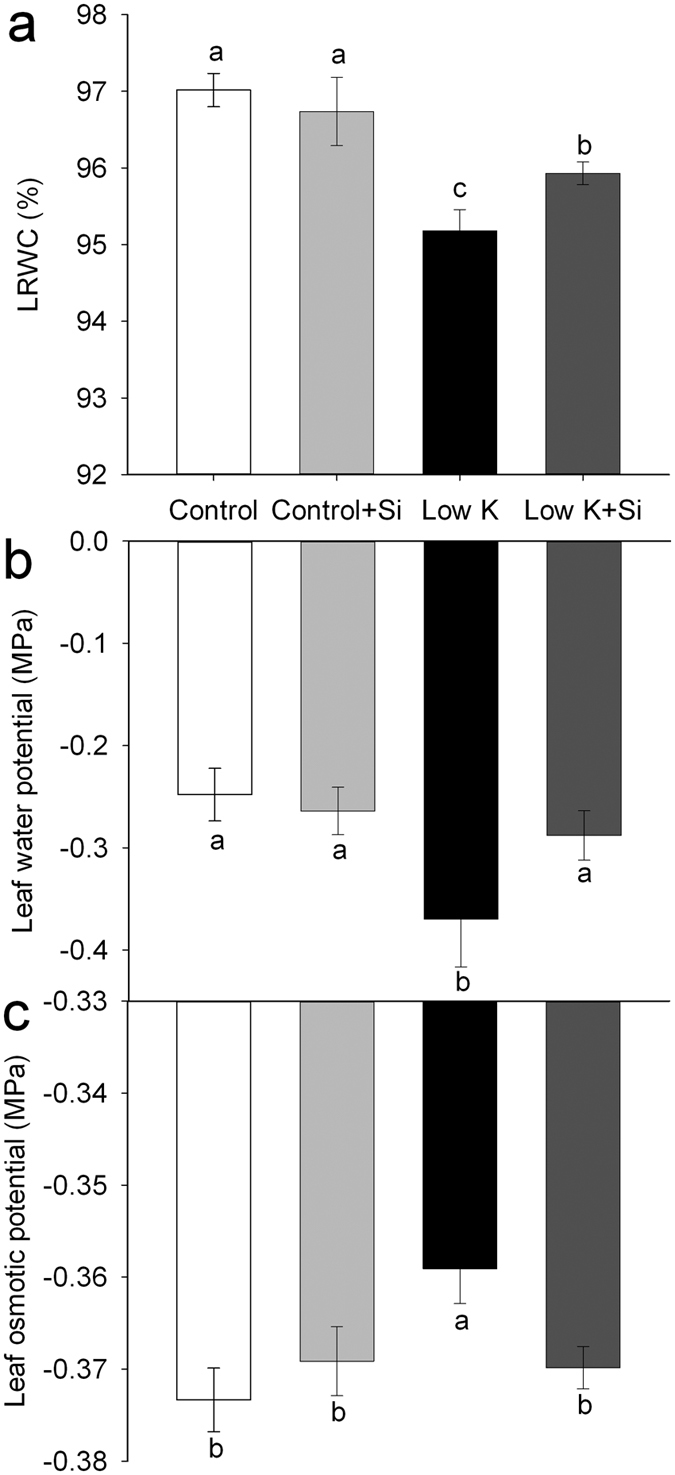
Effects of silicon (Si, 1 mM) on leaf relative water content (LRWC) (**a**), leaf water potential (**b**) and leaf osmotic potential (**c**) of sorghum plants grown under control (3 mM KCl)and low K (0.05 mM KCl) conditions. All parameters were measured after 15 days of treatment. Values are presented as the mean ± SE (n = 5). Different letters in one measure indicate statistically significant differences at *P* < 0.05.

Water status is reflected by the LRWC^15^,^16^. K deficiency increases the plant’s susceptibility to water stress, and plants with an adequate K supply have better hydrated tissues than those with K deficiency^15^,^16^. In this study, **t**he LRWC was decreased after 15 days of K deficiency and Si significantly reduced the K-deficiency-induced decrease in LRWC (Fig. 3a). Leaf water potential also decreased after the 15-day K-deficiency treatment, but Si application maintained the leaf water potential at the same level as that of control (Fig. 3b). These results indicated that Si application improved the plant water-status under K-deficiency conditions. Osmotic potential was significantly enhanced after a 15-day K-deficiency treatment, but Si application eliminated the K-deficiency-induced increase in osmotic potential (Fig. 3c). The lower osmotic potential in Si-treated plants suggested that Si could improve the plant-water status by decreasing the osmotic potential.

### Si application did not enhance the K concentration under the K-deficiency condition

**Figure 4:**
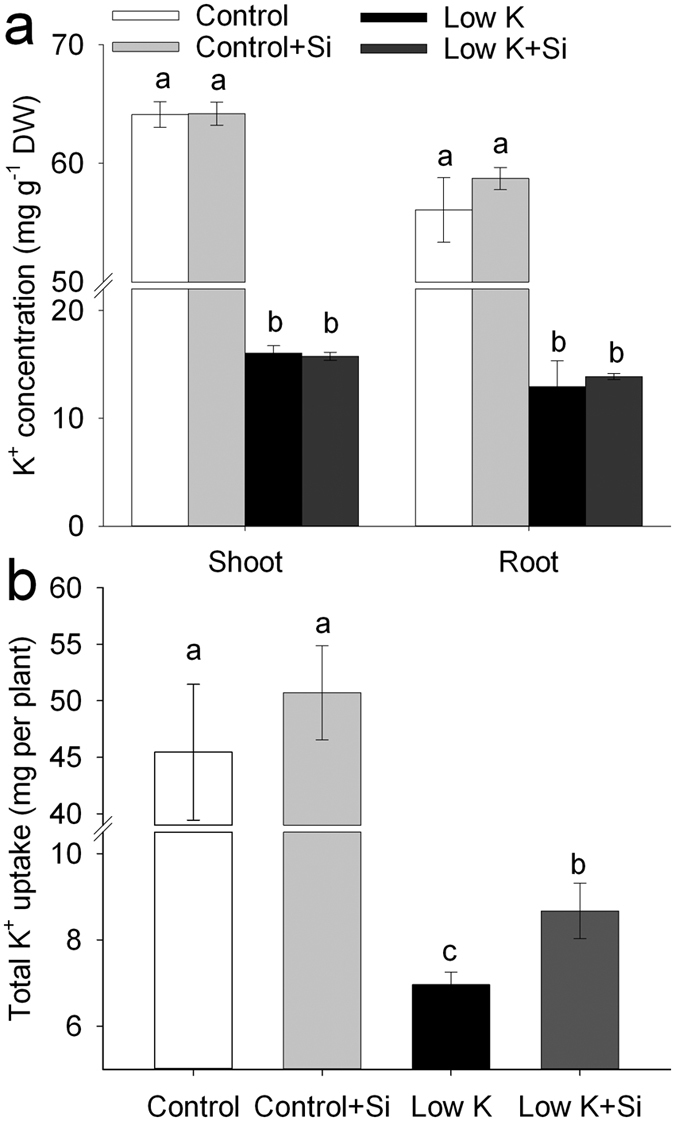
Effects of silicon (Si, 1 mM) on K^+^ concentration (**a**) and total K^+^ uptake (**b**) of sorghum plants grown under control (3 mM KCl)and low K (0.05 mM KCl) conditions. All parameters were measured after 15 days of treatment. Values are presented as the mean ± SE (n = 3). Different letters in one measure indicate statistically significant differences at *P* < 0.05.

Activated K^+^ uptake in plants by the induction of the expression of high-affinity transporters was considered to be a major mechanism of adaptation to K starvation^29^,^30^,^31^. As shown in Fig. 4, the K^+^ concentration in both shoots and roots sharply decreased after 15 days of K deficiency. Si application did not influence the K^+^ concentration in either shoots or roots under control or K-deficiency conditions. Total K^+^ uptake per plant was decreased by K deficiency, and was much lower in plants that did not receive Si treatment (Fig. 4). This result suggested that the increased total K^+^ absorption in Si-treated plants under K-deficiency conditions was the consequence of increase in biomass. The same K^+^ concentration between the Si-treated and -untreated plants indicates that Si alleviating the K deficiency was not due to a direct increase in K^+^ absorption.

### Si improved the plant-water status by maintaining the high level of whole-plant hydraulic conductance and root hydraulic conductance

**Figure 5:**
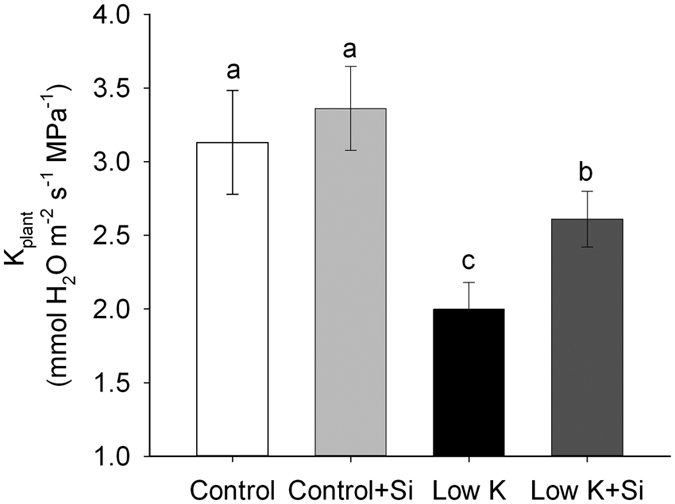
Effects of silicon (Si, 1 mM) on whole-plant hydraulic conductance (K_plant_) of sorghum plants grown under control (3 mM KCl) and low K (0.05 mM KCl) conditions. The K_plant_ was calculated by the transpiration rate determined gravimetrically divided by the difference between culture solution and leaf water potential. Values are presented as the mean ± SE (n = 6). Different letters in one measure indicate statistically significant differences at *P* < 0.05.

The overall plant water uptake is represented by whole-plant hydraulic conductance (K_plant_), which consists of leaf, stem, and root hydraulic conductance^32^. In this study, under normal growth conditions, Si application did not change K_plant_. K deficiency largely decreased K_plant_ and Si application significantly reduced this decrease. K_plant_ in plants treated with Si was 31% (*p* < 0.05) higher than that of plants without Si under K deficient conditions (Fig. 5). These results showed that Si-treated plants had greater water uptake ability than untreated plants.

**Figure 6:**
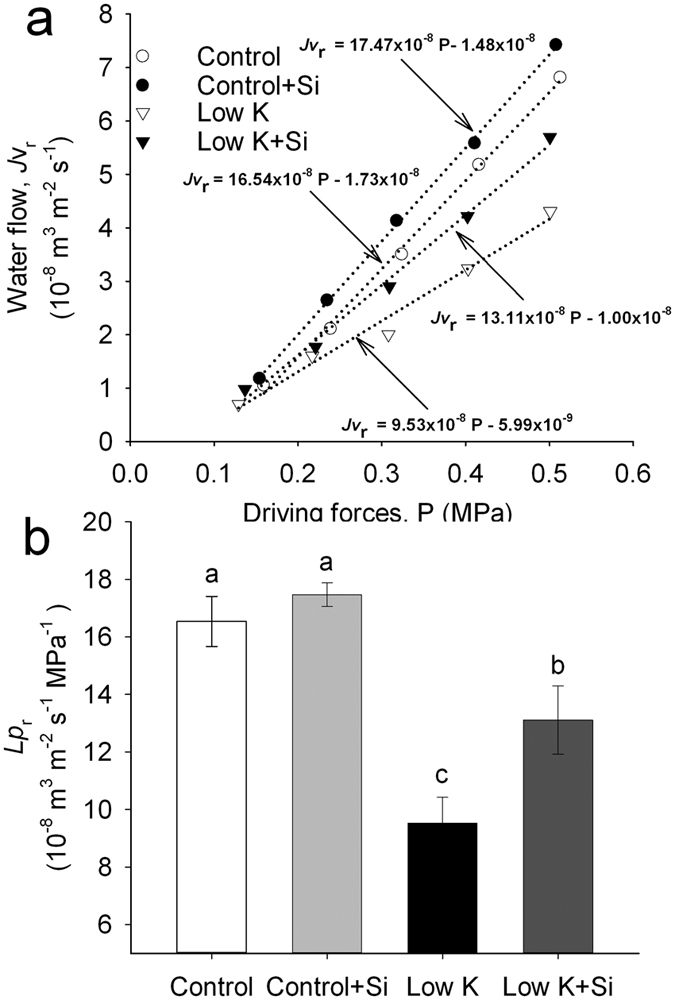
Effects of silicon (Si, 1 mM) on root hydraulic conductance (*Lp*_r_) of sorghum plants grown under control (3 mM KCl) and low K (0.05 mM KCl) conditions. The *Lp*_r_ was measured using a pressure chamber. Exuded sap was collected for 60 s for a given gas pressure and weighed, and the root surface area was then scanned and analyzed. The water flow, *J*v_r_ in m^3^ m^−2^ s^−1^, was obtained and *Lp*_r_ (m^3^ m^−2^ s^−1^ MPa^−1^) is calculated from the slope of *J*v_r_ against the driving force *P*, consisting of *P*_gas_ and an osmotic gradient (**a**). The mean ± SE (n = 6) are shown in (**b**). Different letters in one measure indicate statistically significant differences at *P* < 0.05.

Root hydraulic conductance (*Lp*_r_) is usually the lowest within the liquid component of the soil–plant–air continuum^33^, and roots contribute the majority of the whole-plant hydraulic resistance^34^. In this study, under control conditions, Si application did not change the *Lp*_r_. However, K deficiency significantly decreased *Lp*_r_, and Si application significantly moderated this decrease. *Lp*_r_ in Si-treated plants was 35% (*p* < 0.05) higher than that in untreated plants under the K deficiency conditions (Fig. 6). These results indicated that the higher *Lp*_r_ in Si-treated plants could contribute to the Si-mediated increase in plant water uptake under K-deficiency condition.

### Si alleviated *Lp*_r_ by enhancing aquaporin activity and xylem sap K^+^ concentration

**Figure 7:**
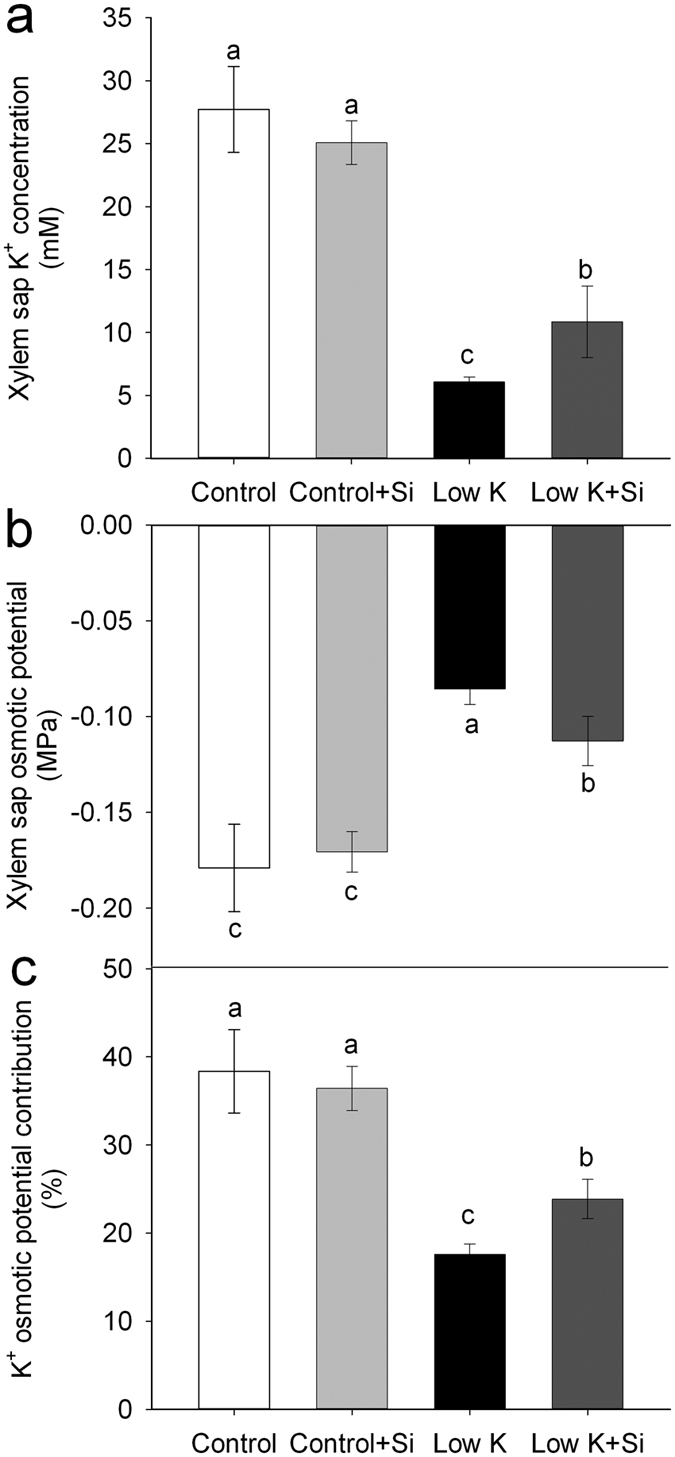
Effects of silicon (Si, 1 mM) on xylem sap K^+^ concentration (**a**), osmotic potential (**b**), and K^+^ osmotic potential contribution (**c**) of sorghum plants grown under control (3 mM KCl) and low K (0.05 mM KCl) conditions. All parameters were measured after 15 days of treatment. Values are presented as the mean ± SE (n = 3). Different letters in one measure indicate statistically significant differences at *P* < 0.05.

In the process of water uptake by roots, water can move radially toward the xylem along three pathways: the apoplastic, symplastic, and transcellular pathways. The symplastic and transcellular pathways are collectively referred to as the “cell-to-cell pathway”^35^,^36^. The cell-to-cell pathway plays an important role in water transport in the roots under certain conditions and is driven by the osmotic gradient between the soil and root xylem sap^34^. In the present study, the K^+^ concentration in xylem sap was largely decreased after 15 days of K deficiency, but Si application significantly reduced the K-deficiency-induced decrease. The xylem sap K^+^ concentration in the Si-treated plant was 80% (*p* < 0.05) higher than that of untreated plants (Fig. 7a). The xylem sap osmotic potential was significantly enhanced after 15 days of K deficiency. However, under K deficiency conditions, the xylem sap osmotic potential of the plant was 32% (*p* < 0.05) lower in Si-treated plants than in untreated plants (Fig. 7b). The low osmotic potential in xylem sap indicates the higher osmotic gradient and driving force between the soil and the root xylem sap in Si-treated plants under the K-deficient condition. The contribution of K^+^ to the xylem sap osmotic potential was also investigated and the result showed that K^+^ accounts for 38% of the osmotic potential under the control condition with or without Si, 24% of the osmotic potential under K-deficiency conditions with Si treatment, and 17% under K-deficiency conditions without Si. The contribution of K^+^ to the osmotic potential in Si-treated plants was 41% (*p* < 0.05) higher than that of in Si-untreated plants under the K-deficiency condition (Fig. 7c).

**Figure 8:**
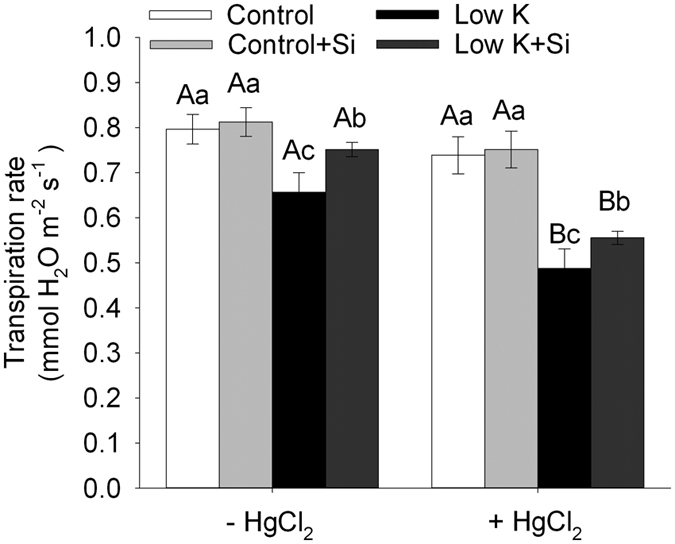
Effect of aquaporin inhibitor (HgCl_2_) on the transpiration rate of sorghum plants with and without Si application grown under control (3 mM KCl) and low K (0.05 mM KCl) conditions. The seedlings were divided in two groups, as follows: one measured the normal transpiration rate, and the other was treated with 50 μM HgCl_2_ for 5 min and then rinsed with distilled water before measuring the transpiration rate in the culture solutions. Values are presented as the mean ± SE (n = 6). Different capital letters and lower cases indicate statistically significant differences at *P* < 0.05 between HgCl_2_ treatments or K and Si treatments.

In addition to the osmotic gradient, it has been reported that the cell-to-cell pathway can be largely controlled by the activity of aquaporin^33^. In several studies, aquaporin activity has been assessed using inhibitors such as mercuric chloride (HgCl_2_), which blocks protein channels and results in a reduction of membrane permeability to water without any adverse effect on the tissue^25^. In this study, **i**n the absence of HgCl_2_, the transpiration of Si-treated plants was higher than that of plants without Si treatment under K-deficient condition (Fig. 8). Under control conditions, the presence of HgCl_2_ decreased the transpiration rate slightly (approximately 6%) in plants that were treated both with and without Si. However, a significant inhibition by HgCl_2_ in plant transpiration (26% decrease, *p* < 0.05) was found under K-deficient condition. It was also found that the transpirations of plants both with and without Si were decreased by HgCl_2_ under the K-deficiency condition, but the differences between the Si-treated and -untreated plants were smaller compared with those before HgCl_2_ addition (Fig. 8). These results showed that aquaporin could be partly responsible for the enhanced *Lp*_r_ under the K-deficiency condition.

### Si application regulated the expression of aquaporins genes and K^+^-related genes

**Figure 9:**
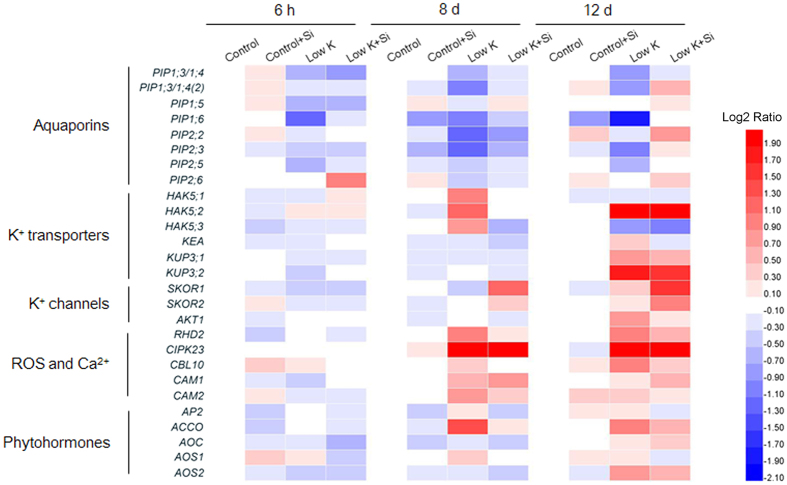
Effects of silicon (Si, 1 mM) on expression of aquaporin genes and putative genes homologous to *Arabidopsis* K^+^-related genes in root of sorghum plants with and without Si application grown under control (3 mM KCl) and low K (0.05 mM KCl) conditions. Each treatment includes three replications and each replication includes three technical replications. Colors indicate change of transcript level in the treated samples with respect to the control samples (red for up-regulation, blue for down-regulation. See color bar at the right of figure).

To investigate the molecular basis for Si alleviation of K-deficiency in plants, we examined the expression levels of aquaporins genes and putative genes homologous to *Arabidopsis* K^+^-related genes as reviewed by Wang and Wu^37^ including those encoding K^+^ transporters, K^+^ channels, regulatory factors, and signaling components in sorghum roots by qRT-PCR (Fig. 9 and Supplementary Table S3). Under control condition, the expression levels of tested genes showed no change by Si application. Under K-deficient condition, mRNA levels of plasma membrane intrinsic protein (*PIP*) *PIP1;3/1;4(2)*, *PIP1;6*, *PIP2;2*, *PIP2;3* decreased up to 2-fold, compared with levels under the control condition, whereas they remained unchanged under K-deficient plus Si treatment. Expression of putative high affinity K^+^ transporter (HAK) gene *HAK5.2*, which is regarded as the marker gene in *Arabidopsis* response to K^+^ deficiency^37^ and another K^+^-deficiency-induced K^+^ transporter K uptake permease (KUP) *KUP3.2* increased by 7.0- and 3.3-fold, respectively, at the 12^th^ day under K-deficient condition, whereas they increased less (4.4- and 2.9- fold) under K-deficient plus Si condition. For the putative sorghum *stelar K*^*+*^ *outward rectifier* (*SKOR)* gene *SKOR1* and *SKOR2*, those are specifically expressed in the root stele (pericycle and stelar parenchyma) and encode the SKOR channel mediating K^+^ secretion from root cortex cells into the xylem^38^, their expression levels were not affected by K-deficient treatment, whereas they increased significantly by 2.8- and 2.1-fold under K-deficient plus Si condition, respectively.

ROS, Ca^2+^, ethylene, and jasmonic acid have been considered the major signals in plant responses to K^+^-deficiency stress^37^. In this study, the putative NADPH oxidase gene *root hair defective 2* (*RHD2)*, which is involved in ROS generation^39^, is significantly up-regulated at the 8^th^ and 12^th^ day under K-deficient condition, whereas the degree of up-regulation was less under K-deficient plus Si condition. Similar changes in mRNA levels were also found in putative sorghum *calcineurin B-like protein* 10 *(CBL10)*, *CBL-interacting serine/threonine-protein kinase 23* (*CIPK23)*, and *calmodulin 2 (CaM2)*, those involved in the perception and transduction of K^+^-deficiency-induced Ca^2+^ signals, *1-aminocyclopropane-1-carboxylate (ACC) oxidase* (*ACCO)*, encoding the ethylene synthesis enzyme ACC oxidase, and *allene oxide synthase (AOS)*, encoding the jasmonic biosynthetic enzyme allene oxide synthase^37^.

The up-regulation of the aquaporins genes by Si application under K-deficient condition could be partly responsible for the enhanced aquaporin activity. The up-regulation of the *SKOR* genes and the down-regulation of the *HAK5* gene could contribute to xylem sap K^+^ concentration under K-deficient condition. Also, Si negatively regulated the Arabidopsis K transporter 1 (AKT1), an inward-rectifying channel, by down regulation of the *CIPK23* gene, and resulted in inhibition of the K^+^ translocation out of xylem. Moreover, the down-regulation of the K^+^-deficiency-responsive genes by Si under K deficient condition suggested that the K deficiency was moderated by Si application, and this moderation was not due to the increase in K uptake by the up-regulation of K^+^ transport proteins at the transcriptional level.

## Discussion

Severe K deficiency first inhibits plant growth, and leaf necrosis appears in old leaves that had a prolonged exposure time^26^. In the present study, Si application significantly inhibited the decrease in total dry weight under K-deficiency condition, and leaf necrosis in old leaves was also alleviated in Si-treated plants compared with Si-untreated plants (Fig.1a, Supplementary Fig. S1), indicating that Si application alleviated the K deficiency. The effect of Si on alleviating the K deficiency was also investigated under serial K concentrations (from 6 mM to 0 mM, see Supplementary Fig. S2). The result showed that Si could only alleviate K deficiency when the K concentration was lower than 1.5 mM, and the biggest effect occurred when the K concentration was set between 0.5 mM and 0.01 mM. The results also showed that Si only plays a role when the plant growth was obviously inhibited by K deficiency. Therefore, Si enhanced plant growth specifically under the condition of K deficiency. It is of note that although Si showed a significant effect on alleviating K deficiency, it cannot entirely eliminate the adverse effect of K deficiency under a severe K-deficiency condition.

The major mechanism of adaptation to K starvation in plants is the activation of K^+^ uptake through the induction of expression of high affinity transporters^29^,^30^,^31^. A previous study showed that Si application enhanced plant K^+^ concentration^4^. However, in this study, the K^+^ concentration in both the root and shoot was not affected by Si application. And consistently, the putative *HAK5* and *AKT1*, which mediate almost all K^+^ absorption in *Arabidopsis* roots^37^, were not up-regulated by Si compared with that without Si application under K deficient condition (Fig. 9). Severe K deficiency was also reported to decrease the photosynthetic rate, and maintenance of a high photosynthetic rate is a base of accumulation of more biomass under K deficiency^21^,^27^,^28^. In this study, although the K concentration in plants that were treated with and without Si was low and at the same level under K deficiency, the photosynthetic rate and dry weight in Si-treated plants were much higher than in those without Si, suggesting that the Si-alleviated K-deficiency-induced growth inhibition was not directly due to the increase in K uptake.

Severe K deficiency generally induces stomatal conductance and lowers transpiration rate, which results in a lower photosynthetic rate^21^,^27^,^28^. In this study, plants treated with Si maintained a higher stomatal conductance and transpiration rate, which is coincident with the higher photosynthetic rate (Fig. 2). These results suggest that the Si-improved plant-water status could be the major reason for the increased photosynthetic rate under the K-deficient conditon. Water status, as reflected by LRWC, is the basis of metabolic activity in tissues^15^,^16^. It is widely accepted that K deficiency increases a plant’s susceptibility to the water stress and that plants with an adequate K supply have better hydrated tissues than those with K deficiency^37^. In this study, K deficiency decreased the LRWC and water potential, which was moderated by Si application. The results of this study suggest that Si alleviates K deficiency by improving plant-water status.

Plant water-status involves the balance between the differences in water uptake through the roots and the transpiration in leaves^15^,^16^. Severe K deficiency has been reported to favor stomatal closure and decrease the transpiration rate^22^,^40^. However, the stomatal conductance and transpiration rates were maintained at higher levels in Si-treated plants compared with untreated plants under K-deficiency treatment. This suggests that Si’s improvement of the plant-water status is a result of an increase in water uptake, but not because of a reduction in water loss.

The overall plant water uptake is represented by whole-plant hydraulic conductance (K_plant_), which consists of leaf, stem and root hydraulic conductance^32^. In this study, K deficiency caused a decrease in K_plant_ that was moderated by Si application (Fig. 5). The seedlings used in the experiment did not form stems. Therefore, K_plant_ was only affected by the root and leaf hydraulic conductance. K_leaf_ was not measured in this study due to technical limitations, but it tends to be similar to leaf water potential^41^. In the present study, the leaf water potential of sorghum with Si was higher than that without Si under K-deficiency condition, suggesting that plants with Si application maintained a higher K_leaf_ than without Si. Root hydraulic conductance (*Lp*_r_) is usually the lowest within the liquid component of the soil–plant–air continuum^33^ and roots contribute the majority of the whole plant hydraulic resistance^34^. In addition, the shoot/root ratio was not affected by Si under the K-deficiency conditon (Fig. 1c). Therefore, the similar extent of Si-enhanced K_plant_ (31%) and *Lp*_r_ (35%) (Figs 5 and 7) indicated that Si enhanced the plant water uptake was mainly ascribed to the enhancement of the *Lp*_r_.

Gas exchange rates and photosynthesis are limited by the hydraulic conductance of the liquid water pathway from the soil to the leaves^42^,^43^,^44^,^45^. In this study, K deficiency caused a significant decrease in the LRWC (Fig. 3). The whole-plant transpiration rate and leaf transpiration was decreased by 18% and 57%, respectively, suggesting that water is a serious limitation for photosynthesis under the K-deficient condition. However, the plant hydraulic conductance was 31% higher in Si-treated plants than Si-untreated plants under the K-deficient condition. Similarly, the whole-plant transpiration rate and leaf transpiration was increased by 14% and 94%, respectively (Figs 2 and 8). Meanwhile, the photosynthesis rate of the leaf was enhanced by 139% in Si-treated plants compared to untreated plants. Although there is not a clear quantitative relationship between the plant water conductance and the growth rate or photosynthetic rate under K deficiency, the highly consistent tendency for plant water conductance, transpiration rate, photosynthesis, and dry weight suggests that the alleviation of the plant-water status through the enhancement of the water hydraulic conductance played an important role in Si-alleviated K deficiency.

*Lp*_r_ represents the root water uptake capacity and it is determined by root surface, root anatomy and root water permeability^46^. In the root water uptake, the cell to-cell pathway can be largely controlled by the activity of aquaporins, which respond relatively rapidly and reversibly, causing changes in *Lp*_r_^33^. In this study, plant transpiration with and without Si is decreased by HgCl_2_, but the difference between Si-treated and -untreated plants was reduced after HgCl_2_ treatment, and aquaporin contributed to 29% of the Si-induced increase in transpiration. Liu *et al.*^24^,^25^ reported that aquaporin regulation accounts for the mechanism of Si-induced alleviation of the plant-water status under drought and salt stress conditions. However, in the present study, 71% of the Si-induced increase in transpiration remained in addition to the aquaporin-regulated water uptake. Thus, other important approaches that mediate the Si-enhanced water uptake under K-deficiency condition must be considered.

The cell-to-cell pathway plays an important role in water transport in the roots under certain conditions, and is driven by the osmotic gradient between the soil and the root xylem sap^34^. In this study, the osmotic potential of root xylem sap was 20% lower in Si-treated plants than in Si-untreated plants (Fig. 7). This resulted in an increase in the osmotic gradient between the soil and the root xylem. The osmotic gradient between the cultivated solution and the xylem in plants treated with Si was −0.092 MPa; it was −0.073 MPa in Si-untreated plants under the K-deficiency condition. Thus, the osmotic gradient was 26% higher in Si-treated plants than Si-untreated plants.

In addition to serving as an important component of the xylem osmotic potential, xylem K^+^ plays a critical role in modifying the xylem hydraulic conductance and water relations in plants in response to ion-mediated volume changes of pectin in pit membranes^47^. Nardini *et al.*^48^ reported that a 30 to 60% increase in stem hydraulic conductance in *Laurus nobilis* is associated with an increase in the xylem sap K^+^, from 3 to 12 mM. In another experiment on laurel plants, short-term K fertilization led to an increase in xylem sap K^+^ and caused a rapid (within 24 h) 45% increase in the transpiration rate and a 30% increase in plant hydraulic conductance^19^. Cation-mediated enhancement of xylem hydraulic conductivity has been reported for both herbaceous and woody species^49^. In this study, the K^+^ concentration in the root xylem sap was 80% higher in Si-treated plants than in untreated plants under K-deficiency condition. Although we cannot estimate the contribution of the enhanced K^+^ concentration to xylem hydraulic conductance and *Lp*_r_, based on the previous study, the huge discrepancy in the xylem sap K concentration between plants treated with and without Si is expected to lead to much higher plant hydraulic conductance and transpiration rates in Si-treated plants.

To date, there is no information available on how Si is involved in regulating important metabolic pathways in plants because of the absence of evidence of organic Si in plant tissue. However, there is some evidence that supports the idea that Si can be involved in regulating the plant stress response. For example, the expression of nearly 4,000 genes was affected in Si-treated *Arabidopsis* that was infected with a fungal pathogen^50^. Liu *et al.*^24^ also suggested that reactive oxygen species (ROS) could participate in the Si-regulated aquaporin activity under salt stress. In this study, the aquaporins genes were up-regulated by Si application under K-deficient condition, which could be partly responsible for the enhanced aquaporin activity (Fig. 9 and Supplementary Table S3). The *SKOR* genes mediate K^+^ secretion from root cortex cells into the xylem. Pilot *et al.* reported that the level of *SKOR* transcripts decreased after K^+^ shortage in Arabidopsis^51^. However, other studies showed that SKOR did not transcriptionally respond to K^+^ deprivation^52^, and also short-term K^+^ resupply after long-term K^+^ starvation in Arabidopsis^53^. In addition, *OsSKOR* gene was up-regulated under low-K^+^ stress^31^. These inconsistent results suggest that transcriptional response of *SKOR* to K^+^ deficiency may differ among different species and K^+^ deficiency treatments. In this study, the expression level of *SKOR* showed no change under low K condition, but the it was up-regulated by Si application under K-deficient condition. In addition, the *HAK5*, *CIPK23* and *RHD2* genes were down-regulated by Si application under K-deficient condition. Those changes contribute to xylem sap K^+^ concentration under K-deficient condition (Fig. 9 and Supplementary Table S3). Therefore, it appears that Si can regulate the K-deficiency responses at the transcriptional level directly or indirectly. Recently, the K-deficiency response signals have been clarified in model plants, such as *Arabidopsis*. In particular, it has been shown that ROS, Ca^2+^, ethylene, and jasmonic acid are involved in plant signaling responses to K^+^ deficiency^37^,^54^,^55^,^56^,^57^. Although no previous studies have reported how Si is involved in plant signaling regulation under various stresses, the application of Si that influences the ROS generation and elimination has been widely reported^24^,^58^. In this study, genes related to ROS metabolism, Ca^2+^ signal perception, and ethylene and jasmonic acid synthesis are down-regulated by Si application under K-deficient condition (Fig. 9 and Supplementary Table S3). Thus, it appears that Si can also influence the plant signaling regulation under K-deficient stress. Therefore, although we do not yet have a clear picture of how Si regulates the K concentration in xylem under K deficiency, based on the present and previous studies, we propose the following model: under the K-deficient condition, Si directly or indirectly influence the plant signaling regulation, which are involved in regulating the K channels and transporter gene expression and post-transcriptional regulation. The regulation of K channels and transporters, especially the outward-rectifying channel SKOR, which mediate K concentration in the xylem under the K-deficient condition, may lead to enhanced K concentration in the xylem.

## Conclusion

**Figure 10:**
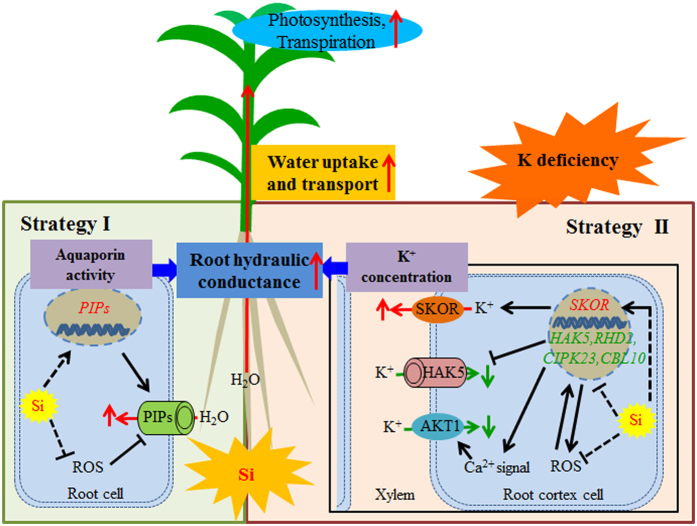
A schematic model for silicon (Si) moderated the K deficiency in sorghum by improving the water uptake and transport. Two strategies are involved in Si enhance the root hydraulic conductance under K-deficient condition. For strategy I, Si could enhance the aquaporin activity by both up-regulating the expression of aquaporin genes (*PIPs*) and alleviating the decrease of aquaporin activity caused by ROS (Reactive Oxygen Species). For strategy II, Si could activate the K^+^ translocation to xylem by the activation the expression of *SKOR* gene, which mediate K^+^ secretion from root cortex cells into the xylem; Si could also down-regulate the expression of *HAK5* gene and may interact with Ca^2+^ and ROS signals to negatively regulate the AKT1 channel to inhibit the K^+^ translocation out of xylem (red for up-regulation, green for down-regulation). The enhanced aquaporin activity and K^+^ concentration in xylem resulted in increased root hydraulic conductance and higher whole-plant hydraulic conductance, which contributed to an increase in water uptake and transport. The improved water uptake and transport alleviates the K-deficiency-induced plant dehydration and moderates the K deficiency.

K is widely involved in regulating the plant-water status, and severe K deficiency induces plant dehydration. In this study, Si application alleviated the K deficiency while maintaining higher relative water content, water potential, transpiration and plant hydraulic conductance. Combining the present findings with those of previous studies, a model describing how Si is involved in alleviating K deficiency by moderating plant-water status was proposed (Fig. 10). (1) Under the K-deficient condition, Si activated the expression of *SKOR*, down-regulated the expression of *HAK5* and negatively regulated the AKT1 channel, which resulted in the concentrating of K in xylem. (2) The higher K concentration in the xylem sap contributed to the increase in the osmotic gradient and the xylem hydraulic conductance, leading to enhanced root hydraulic conductance. (3) Si could also participate in regulating the aquaporin activity, resulting in increased root hydraulic conductance. (4) The higher root hydraulic conductance resulted in higher whole-plant hydraulic conductance, which contributed to an increase in water uptake and transport, resulting in an alleviation of K-deficiency-induced plant dehydration, and alleviating the K deficiency. However, the K^+^-related genes, whose product are almost all hypothetical proteins, used for expression analysis were gotten from homology searching based on information in *Arabidopsis*, rice and maize. Furthermore, besides transcriptional regulation, posttranscriptional regulation also plays a critical role in plant response to K deficiency, especially for the K^+^ channels. Thus, the mechanisms underlying the Si-mediated K^+^ concentration in the xylem require further study.

## Materials and Methods

### Seedling cultivation and Si and K deficiency treatment

Sorghum cultivar (*Sorghum bicolor* [L.] Moench. cv. Gadambalia) was used in this study. This cultivar can uptake a large amount of Si, and Si application can improve its water status under drought and salt stress conditions^24^,^25^. The seedlings were cultivated in a growth chamber that was set to a 14/10 h day/night cycle at a day/night temperature of 28/25 °C with 40 to 50% relative humidity. The amount of photosynthetically active radiation (PAR) to the upper plant was 600 μmol m^−2^ s^−1^.

Sterilized seeds were germinated in an incubator at 24 °C under dark conditions. After germination for 4 days, uniform seedlings (6 ± 0.5 cm)were selected and transplanted into a plastic container (40 × 28 × 14 cm) with 8 liters of one-quarter strength Hoagland culture solution with no KNO_3_, NO_3_^−^ was provided by Ca(NO_3_)_2_ and the K was supplied by KCl. The experiment included four treatments: control, control + Si, low K and low K + Si. The K concentration was 0.05 mM in the low K (K deficiency) treatments and 3 mM in the control (K sufficiency) treatment, supplied by KCl. Under 3 mM K concentration, the growth of seedlings showed no difference compared to that under 6 mM K (normal K concentration in Hoagland solution). The growth of seedlings showed obvious inhibition under the 0.05 mM K condition (Supplemental Fig. S1). The pH of the culture solution was adjusted to 6.0^53^. Si was set to 0 mM in the without Si treatment and 1mM H_2_SiO_3_ in the Si treatments. H_2_SiO_3_ was produced by passing sodium silicate solution through a column filled with cation-exchange resins^58^. The culture solution was renewed every three days and continuously aerated. Fifteen days after transplantation, samples were collected and the parameters listed below were measured.

### Dry weight and root/shoot ratio

Fifteen days after transplantation, the plant was harvested. The shoots and roots were separated and dried at 75 °C for 72 h. The total weight and root/shoot ratio were calculated. Each treatment included 10 plants.

### Photosynthetic rate, stomatal conductance and transpiration rate

Fifteen days after transplantation, the upper fully expanded leaves were selected to measure the photosynthetic rate, stomatal conductance and transpiration rate using a portable photosynthesis system (Li-6400; LI-COR Inc., Lincoln, NE, USA) between 10:00 AM and 12:00 AM. The leaf was placed in a 6 cm^2^ chamber at a photo flux density of 1000 μmol m^−2^s^−1^. Each treatment included six plants.

### Leaf relative water content, leaf water potential and osmotic potential

Fifteen days after transplantation, the upper fully expanded leaves were sampled and LRWC was measured and calculated according to Liu *et al.*^25^. Upper fully expanded leaves were covered with aluminum foil before excision from the plant and the water potential was measured using a pressure chamber (Model 3500, Soil Moisture Corp., Santa Barbara, CA, USA). The leaf osmotic potential of the upper fully expanded leaves was measured using a dew point microvolt meter (Model 5600, Wescor, Logan, UT, USA). Each treatment included five replications.

### K^+^ concentration and total K^+^ uptake in the tissue

Dried shoot and root samples were ground to powder, weighed and then digested using nitric acid at 320 °C for 5 h. K^+^ concentrations were measured using an atomic adsorption spectrometer with a flame photometer (ZL5100, PerkinElmer, Inc., USA)^59^. The K^+^ absorption was calculated based on the dry weight and tissue K^+^ concentration. Each treatment included three replications.

### Whole-plant hydraulic conductance

K_plant_ was calculated according to the following equation^32^:


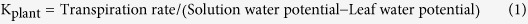


The transpiration rate was determined gravimetrically. The leaf water potential was measured as described above. In hydroponic culture, the culture solution water potential was −0.032 MPa in the control, −0.034 MPa in control + Si, −0.018 MPa in low K, and −0.019 MPa in low K + Si treatment groups. Each treatment included six replications.

### Transpiration rate in response to aquaporin inhibitor

Aquaporin-mediated water transport was investigated by measuring changes in the transpiration rate in response to an aquaporin inhibitor (HgCl_2_), according to Knipfer *et al.*^60^. The seedlings were divided into two groups: one group (6 plants) was used for measuring the transpiration rate, and the other group (6 plants) was treated with 50 μM HgCl_2_ for 5 min and then rinsed with distilled water (devoid of HgCl_2_) before measuring the transpiration rate in the culture solutions. The transpiration rate was determined gravimetrically.

### Root hydraulic conductance

*Lp*_r_ based on the root surface area was measured using a pressure chamber (Model 3500, Soil Moisture Corp., Santa Barbara, CA, USA), according to the method of Miyamoto *et al.*^61^. The roots were enclosed in a steel chamber and pneumatic pressure was applied to the root medium. The pressure in the chamber was increased in steps of 0.1 MPa up to a pressure of 0.5 MPa. Exuded sap was collected for 60 s at a given gas pressure and weighed. The root surface area was then scanned and analyzed using WinRHIZO PRO 2009 software (Regent Inc., Canada). The water flow, *J*v_r_ in m^3^ m^−2^ s^−1^, was obtained. The osmotic potential of the collected exuded sap was measured as described above and *Lp*_r_ (m^3^ m^−2^ s^−1^ MPa^−1^) was calculated from the slope of *J*v_r_ against driving force P, consisting of *P*_gas_ and the osmotic gradient, according to the following equation:


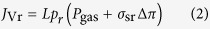


In this equation, σ_sr_ is the root reflection coefficient for nutrient salts in the xylem, which was estimated to be σ_sr_ = 0.4, according to Miyamoto *et al.*^56^. Each treatment included six plants.

### Xylem sap K^+^ concentration and osmotic potential

Xylem sap was extracted between 10:00 AM and 12:00 AM according to Liu *et al.*^25^. Plants were rapidly cut using a razor blade at the base of the root system. The roots were enclosed in a steel chamber and the surface of the cut was washed with distilled water and blotted dry to remove any disrupted and residual cell. The pneumatic pressure applied to the root medium in the chamber was increased to 0.2 MPa until xylem sap flowed out from the excised tip. The first droplet was discarded to avoid contamination, and then 50 μl of sap was collected from each plant to determine the xylem sap K^+^ concentration and osmotic potential. The xylem sap osmotic potential was measured using a dew point microvolt meter and the ion concentration was measured by an atomic adsorption spectrometer with a flame photometer as described above. Each treatment included three replications.

### Gene expression analysis

Roots were sampled at the 8^th^ day (visible K^+^ starvation symptoms appeared) and the 12^th^ day (significant differences between the plants with or without Si application under K deficiency appeared) after transplantation. In addition, 12 days after transplantation, one group of seedlings (6 plants) under control and control + Si conditions were transplanted to low K and low K+ Si conditions, respectively; roots were sampled also after K deficiency treatment for 6 hours to investigate the short-term starvation responses. The expression of 8 sorghum plasma membrane intrinsic protein (*PIP*) aquaporin genes according to Liu *et al.*^25^ and 19 putative genes homologous to *Arabidopsis* K^+^-related genes based on information in *Arabidopsis*, rice (*Oryza sativa*) and maize (*Zea mays*), were analyzed using quantitative RT-PCR. RNA was isolated and gene expression was analyzed using *Actin1* as constitutive controls^25^. Each treatment included three replications and each replication included three technical replications. The genes and the sequences of their specific primers are listed in Supplemental Table S1. The protein sequence similarities between putative K^+^-related genes and those in *Arabidopsis*, rice and maize are listed in Supplemental Table S2.

### Statistical analysis

Data were analyzed using an analysis of variance (ANOVA) using the Statistical Analysis System (SAS version 8.0) software. The differences between the means were compared using the Tukey-Kramer test (*p* < 0.05). All experiments were repeated at least three times. All plots were created using SigmaPlot12.0 and the different letters indicating statistically significant differences at *P* < 0.05 are indicated in the figures.

## Additional Information

**How to cite this article**: Chen, D. *et al.* Silicon moderated the K deficiency by improving the plant-water status in sorghum. *Sci. Rep.* **6**, 22882; doi: 10.1038/srep22882 (2016).

## Supplementary Material

Supplementary Information
