# Supplementary material for: Silicon moderated the K deficiency by improving the plant-water status in sorghum
Source: Sci Rep. 2016 Mar 10;6:22882. doi: 10.1038/srep22882 (PMC4785406; doi:10.1038/srep22882)
Supplement: Supplementary Information [file srep22882-s1.pdf]

Supplementary Material：

Silicon moderated the K deficiency by improving the plant-water status in sorghum

Daoqian Chen[†], Beibei Cao [†], Shiwen Wang*, Peng Liu, Xiping Deng, Lina Yin, Suiqi Zhang

* **Correspondence:**

Dr. Shiwen Wang

State Key Laboratory of Soil Erosion and Dryland Farming on the Loess Plateau

Institute of Soil and Water Conservation, Northwest A&F University

Xinong Road No. 26,

Yangling, Shaanxi, China. 712100.

shiwenwang@nwsuaf.edu.cn

Supplementary Fig. S1

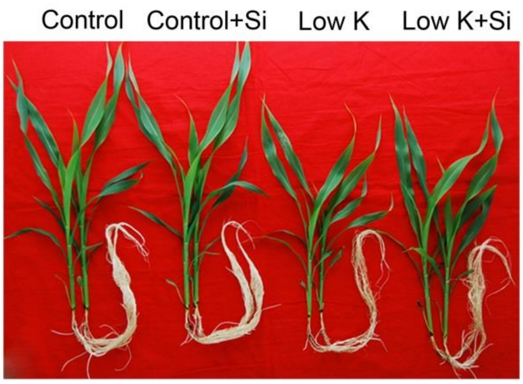

Fig. S1 Effects of silicon (Si, 1 mM) on the plant growth and phenotype of sorghum plants grown under control (3 mM KCl) and low K (0.05 mM KCl) conditions.

Supplementary Fig. S2

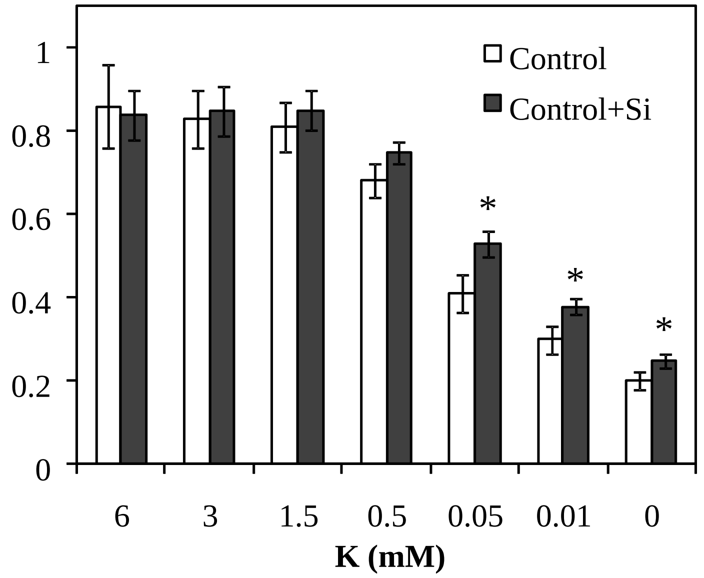

Fig. S2 Effects of silicon (Si, 1 mM) on the plant dry weight of sorghum plants grown under serial K concentration conditions. The dry weight was measured after 15 days of treatment. Values are presented as the mean ± SE (n=10). Asterisk indicate statistically significant differences between Si treatments at $P<0.05$.

Table S1 Genes and oligonucleotides used in real-time quantitative PCR.

**Gene**	**Loci**	**Primer(5′-3′)**
Actin1	Sb01g010030	TGTTCCCTGGGATTGCTG
		GCCGGACTCATCGTACTCA
PIP1;3/1;4	Sb06g025150	AATCGGGTTCGCGGTGTT
		CCAGGCATGGTTCTGGTTGTA
PIP1;3/1;4 (2)	Sb04g032430	GTGGAGCTGGAGTGGTGAA
		GCAAGGATAGGAACATGGGAGT
PIP1;5	Sb04g037800	TTTCGCCGTCTTCCTCGTC
		GGTCGTTCCATGCGTTGG
PIP1;6	Sb10g007610	TGACGGTGCTGACGGTGAT
		GGAGGAGCCCGAAGGTGAC
PIP2;2	Sb02g010760	GACTCCCACGTCCCGGTTCT
		CCCAGGGCTTGTCCTTGTTGT
PIP2;3	Sb04g026650	CCGTGACCTTCGGTTTGTTC
		GCACGTAGTAGGCGCTCTGG
PIP2;5	Sb06g022840	TCGCGGTGTTCATGGTCC
		TCCCAGGTCTTGTCGTTGTTGT
PIP2;6	Sb02g010800	CTTCCGATTGGATTCGCTGTG
		CGGAGGACGATCTGGTGGTA
HAK5.1	Sb03g044790	TCATTTTACAGCCCAGGAGGA
		TGCCTCTTGAACTCTGATGGA
HAK5.2	Sb06g014930	TCTACTCCAGCACTTTCCCC
		CTCCATTGTCGTTGGCGTAG
HAK5.3	Sb04g021210	AGTGAGTGGGATCAGGGAGA
		GCACCGAGAAGAGAAGGAAC
KEA5	Sb01g048670	GCCTCCCATCTCCATCTTGT
		AACCAACGCATAAGGATGCC
KUP3.1	Sb03g045180	TACGGGGACCTAAGCACATC
		ACACCGTCTCCTCATCTTGG
KUP3.2	Sb02g042430	GTTGTGCTTGTTGGCCTCTT
		AGCCAGAACACAACAATCGG
SKOR1	Sb10g008960	ACTGAGCGGTTTAGGGACAA
		GCTGCTCTCATACTGCAACC
SKOR2	Sb06g017830	CCACGGGAGAAGATGGATCA
		CCTGACTGTGTGTGGTTGTG
AKT1	Sb03g029520	TTTTGCTGTCCACTGTTCGG
		CCCCGCTGATGAAAATCTGG
RHD2	Sb05g020380	GCAGACCTTCTTCGACATGG
		CGTACTCCTCCGCTTGATCT
CIPK23	Sb02g003030	CCCATCCTGGTTCTCCACAA
		CAAATCTGGGCGGCTGATAC
CBL10	Sb08g003930	TCGAGCGTGAAGAGGTGAAA
		CAGCGTCCTCAAATGTCTTGT
CAM1	Sb01g037010	TGGAAGGAGGAAACCGTTCA
		GCAGCAGAGGGACTCAAATG
CAM2	Sb05g002010	CGGGCAGATCAACTACAACG
		GATTGTACAGGAGGGGCAGA

AP2	Sb02g039300	GCGTCAACATTGGAGGTCTG
		GATCCTTGAGCTGTTGCGTC
ACCO	Sb10g022640	TCTGTAGGCATCAAGGTGGC
		GCATCAGTGTGTTCCCTGAG
AOC	Sb01g031870	CGTGTACGAGATCAACGAGC
		ACCTTGTTGGTGAAGGGGAC
AOS1	Sb01g007000	GACCATCACCTCGCTCAAGA
		GACAGACATGGACAACGCAG
AOS2	Sb01g042270	GGACGACCGAATTTGAGCAG
		CACAAGCTCTCTCGCAAGTC

Table S2 The protein sequence similarities between putative K^+-related genes and that in Arabidopsis and rice/maize.

Gene	Full name	Loci	Description	Homologous gene in arabidopsis			Homologous gene in rice/maize	
				Gene	Gene ID	Similarity	Gene	Similarity
HAK5.1	*high affinity K^+ transporter 5.1*	Sb03g044790	hypothetical protein	*HAK5*	AT4G13420	55%	*OsHAK5*	82%
HAK5.2	*high affinity K^+ transporter 5.2*	Sb06g014930	hypothetical protein	*HAK5*	AT4G13420	55%	*OsHAK5*	56%
HAK5.3	*high affinity K^+ transporter 5.3*	Sb04g021210	hypothetical protein	*HAK5*	AT4G13420	53%	*OsHAK5*	55%
KEA5	*K^+ efflux antiporter 5*	Sb01g048670	hypothetical protein	*KEA5*	AT5G51710	72%		
KUP3.1	*K uptake permease 3.1*	Sb03g045180	hypothetical protein	*KUP3*	AT3G02050	68%	*ZmHAK2*	96%
KUP3.2	*K uptake permease 3.2*	Sb02g042430	hypothetical protein	*KUP3*	AT3G02050	66%	*ZmHAK7-like*	93%
SKOR1	*stelar K^+ outward rectifier 1*	Sb10g008960	hypothetical protein	*SKOR*	AT3G02850	70%	*OsKOR1*	84%
SKOR2	*stelar K^+ outward rectifier 2*	Sb06g017830	hypothetical protein	*SKOR*	AT3G02850	59%	*OsKOR2*	82%
AKT1	*Arabidopsis K transporter 1*	Sb03g029520	hypothetical protein	*AKT1*	AT2G26650	64%	PREDICTED: *ZmAKT1-like*	95%
RHD2	*root hair defective 2*	Sb05g020380	hypothetical protein	*RHD2*	AT5G51060	63%	*ZmRbohD*	90%
CIPK23	*CBL-interacting serine/threonine-protein kinase 23*	Sb02g003030	hypothetical protein	*CIPK23*	AT1G30270	82%	*OsCIPK23*	95%
CBL10	*calcineurin B-like protein 10*	Sb08g003930	hypothetical protein	*CBL10*	AT4G33000	58%	*OsCBL10*	81%
CAM1	*calmodulin 1*	Sb01g037010	hypothetical protein	*CaM*	AT2G41100	57%	*ZmCaM1*	99%
CAM2	*calmodulin 2*	Sb05g002010	hypothetical protein	*CaM*	AT2G41100	49%	*ZmCaM*	95%
AP2	*apetala 2*	Sb02g039300	hypothetical protein	*AP2*	AT1G72360	33%		
ACCO	*ACC oxidase*	Sb10g022640	hypothetical protein	*ACC oxidase*	AT2G19590	59%	PREDICTED: *ACC oxidase 1*	90%
AOS1	*allene oxide synthase 1*	Sb01g007000	hypothetical protein	*AOS*	At5g42650	59%	PREDICTED: *ZmAOS1*	97%
AOS2	*allene oxide synthase 2*	Sb01g042270	hypothetical protein	*AOS*	At5g42650	57%	*ZmAOS1*	93%
AOC	*allene oxide cyclase*	Sb01g031870	hypothetical protein	*AOC*	At3g25760	66%	*ZmAOC*	86%

Table S3 Effects of silicon (Si, 1 mM) on expression of aquaporin genes and putative genes homologous to Arabidopsis K^+-related genes in root of sorghum plants with and without Si application grown under control (3 mM KCl) and low K (0.05 mM KCl) conditions. Each treatment includes three replications and each replication includes three technical replications. Values are presented as the mean ± SE.

	6 h				8 d				12 d			
	Control	Control+Si	Low K	Low K+Si	Control	Control+Si	Low K	Low K+Si	Control	Control+Si	Low K	Low K+Si
PIP1.3/1.4	1.0±0.02	1.1±0.12	0.7±0.02	0.6±0.03	1.0±0.02	0.9±0.09	0.6±0.04	0.9±0.04	1.0±0.10	1.1±0.10	0.6±0.21	0.9±0.11
PIP1.3/1.4(2)	1.0±0.05	1.2±0.10	0.9±0.02	0.8±0.02	1.0±0.08	0.8±0.05	0.5±0.02	0.8±0.02	1.0±0.06	1.1±0.07	0.6±0.08	1.6±0.10
PIP1.5	1.0±0.02	1.1±0.03	0.7±0.03	0.6±0.03	1.0±0.15	1.2±0.05	0.9±0.02	1.1±0.05	1.0±0.03	1.1±0.05	1.0±0.10	1.2±0.06
PIP1.6	1.0±0.01	1.0±0.03	0.4±0.02	0.9±0.06	1.0±0.02	0.6±0.04	0.5±0.05	0.7±0.07	1.0±0.02	0.6±0.04	0.3±0.09	0.9±0.09
PIP2.2	1.0±0.05	1.1±0.10	0.9±0.06	1.0±0.03	1.0±0.04	0.9±0.01	0.4±0.02	0.6±0.02	1.0±0.02	1.4±0.02	0.9±0.05	1.6±0.05
PIP2.3	1.0±0.01	0.9±0.04	0.7±0.04	0.7±0.05	1.0±0.04	0.7±0.01	0.4±0.02	0.7±0.02	1.0±0.02	0.8±0.01	0.5±0.01	1.2±0.07
PIP2.5	1.0±0.01	1.1±0.02	0.7±0.01	0.9±0.02	1.0±0.05	0.9±0.02	0.8±0.03	0.8±0.03	1.0±0.04	1.0±0.02	0.7±0.17	1.0±0.08
PIP2.6	1.0±0.10	1.0±0.20	1.0±0.10	2.1±0.31	1.0±0.03	1.2±0.03	0.7±0.04	0.9±0.13	1.0±0.06	1.2±0.03	0.9±0.08	1.2±0.19
HAK5.1	1.0±0.03	0.9±0.07	0.5±0.02	1.1±0.05	1.0±0.17	1.0±0.05	1.9±0.14	1.0±0.14	1.0±0.07	0.9±0.03	0.9±0.12	0.9±0.08
HAK5.2	1.0±0.04	0.9±0.07	1.0±0.05	1.1±0.06	1.0±0.13	0.9±0.03	2.4±0.81	1.0±0.04	1.0±0.05	1.0±0.05	7.0±0.55	4.4±0.09
HAK5.3	1.0±0.12	0.8±0.07	0.8±0.07	0.9±0.08	1.0±0.11	1.0±0.18	1.8±0.30	0.7±0.08	1.0±0.12	1.0±0.03	0.6±0.07	0.5±0.07
KEA	1.0±0.01	0.8±0.02	0.9±0.04	1.0±0.02	1.0±0.04	0.9±0.02	0.8±0.05	0.8±0.01	1.0±0.04	1.0±0.18	1.3±0.08	0.9±0.02
KUP3.1	1.0±0.03	1.0±0.04	0.8±0.02	0.8±0.04	1.0±0.04	0.9±0.04	0.9±0.02	0.9±0.02	1.0±0.03	1.0±0.02	1.8±0.09	1.5±0.08
KUP3.2	1.0±0.05	1.1±0.11	0.8±0.06	1.0±0.06	1.0±0.07	0.9±0.04	0.9±0.06	0.9±0.03	1.0±0.01	0.9±0.04	3.3±0.27	2.9±0.49
SKOR1	1.0±0.02	0.9±0.08	0.8±0.08	0.8±0.03	1.0±0.01	1.0±0.02	0.8±0.10	2.2±0.02	1.0±0.05	0.9±0.02	1.4±0.02	2.8±0.10
SKOR2	1.0±0.03	1.1±0.18	0.8±0.07	0.9±0.01	1.0±0.03	0.9±0.02	1.0±0.05	1.3±0.14	1.0±0.10	1.1±0.04	1.2±0.38	2.1±0.16
AKT1	1.0±0.05	0.8±0.05	0.9±0.02	0.9±0.04	1.0±0.14	0.8±0.02	1.0±0.04	0.9±0.03	1.0±0.02	1.0±0.20	1.6±0.07	1.1±0.06
RHD	1.0±0.10	0.7±0.07	1.0±0.08	0.9±0.03	1.0±0.02	0.9±0.11	2.1±0.15	1.1±0.14	1.0±0.09	1.0±0.02	1.9±0.05	1.5±0.19
CIPK	1.0±0.06	1.0±0.01	1.0±0.09	1.0±0.05	1.0±0.02	1.1±0.03	6.0±0.56	4.1±0.67	1.0±0.01	0.9±0.03	27.8±3.68	14.6±1.38
CBL10	1.0±0.02	1.2±0.06	1.1±0.02	1.0±0.02	1.0±0.01	1.0±0.02	1.3±0.07	1.1±0.06	1.0±0.01	1.2±0.05	1.9±0.21	1.3±0.03
CAM1	1.0±0.04	0.9±0.04	0.8±0.05	1.0±0.04	1.0±0.09	1.0±0.05	1.4±0.05	1.8±0.27	1.0±0.02	1.0±0.04	1.2±0.13	1.4±0.20
CAM2	1.0±0.10	1.1±0.07	0.9±0.08	0.9±0.03	1.0±0.02	1.0±0.11	1.7±0.05	1.3±0.24	1.0±0.09	1.4±0.02	1.3±0.05	1.1±0.19
AP2	1.0±0.06	0.8±0.20	0.9±0.03	0.9±0.03	1.0±0.11	0.7±0.08	1.2±0.03	0.7±0.03	1.0±0.05	1.2±0.06	1.1±0.28	0.9±0.10
ACCO	1.0±0.06	0.7±0.05	1.0±0.04	0.9±0.02	1.0±0.01	0.8±0.04	2.7±0.18	1.1±0.01	1.0±0.09	1.0±0.01	2.0±0.21	1.5±0.06
AOC	1.0±0.10	0.8±0.03	0.9±0.03	0.7±0.14	1.0±0.02	0.7±0.05	0.9±0.01	0.8±0.03	1.0±0.10	1.0±0.02	1.2±0.04	1.3±0.12
AOS1	1.0±0.06	1.2±0.08	1.1±0.02	0.8±0.02	1.0±0.04	0.9±0.01	1.3±0.13	1.0±0.08	1.0±0.07	1.2±0.04	1.2±0.03	0.9±0.09
AOS2	1.0±0.04	0.8±0.03	0.8±0.11	0.7±0.07	1.0±0.02	0.9±0.01	0.9±0.04	0.7±0.03	1.0±0.02	0.9±0.02	1.7±0.27	1.6±0.03
